# Power Analysis for Population-Based Longitudinal Studies Investigating Gene-Environment Interactions in Chronic Diseases: A Simulation Study

**DOI:** 10.1371/journal.pone.0149940

**Published:** 2016-02-22

**Authors:** Jinhui Ma, Lehana Thabane, Joseph Beyene, Parminder Raina

**Affiliations:** 1 Department of Clinical Epidemiology and Biostatistics, McMaster University, Hamilton, Ontario, Canada; 2 McMaster University Evidence-based Practice Center, Hamilton, Ontario, Canada; 3 Biostatistics Unit, St. Joseph's Healthcare Hamilton, Hamilton, Ontario, Canada; 4 Centre for Evaluation of Medicines, St Joseph’s Healthcare Hamilton, Ontario, Canada; 5 Population Health Research Institute, Hamilton Health Sciences, Hamilton, Ontario, Canada; University of New South Wales, AUSTRALIA

## Abstract

Conventional methods for sample size calculation for population-based longitudinal studies tend to overestimate the statistical power by overlooking important determinants of the required sample size, such as the measurement errors and unmeasured etiological determinants, etc. In contrast, a simulation-based sample size calculation, if designed properly, allows these determinants to be taken into account and offers flexibility in accommodating complex study design features. The Canadian Longitudinal Study on Aging (CLSA) is a Canada-wide, 20-year follow-up study of 30,000 people between the ages of 45 and 85 years, with in-depth information collected every 3 years. A simulation study, based on an illness-death model, was conducted to: (1) investigate the statistical power profile of the CLSA to detect the effect of environmental and genetic risk factors, and their interaction on age-related chronic diseases; and (2) explore the design alternatives and implementation strategies for increasing the statistical power of population-based longitudinal studies in general. The results showed that the statistical power to identify the effect of environmental and genetic risk exposures, and their interaction on a disease was boosted when: (1) the prevalence of the risk exposures increased; (2) the disease of interest is relatively common in the population; and (3) risk exposures were measured accurately. In addition, the frequency of data collection every three years in the CLSA led to a slightly lower statistical power compared to the design assuming that participants underwent health monitoring continuously. The CLSA had sufficient power to detect a small (1<hazard ratio (HR)≤1.5) or moderate effect (1.5< HR≤2.0) of the environmental risk exposure, as long as the risk exposure and the disease of interest were not rare. It had enough power to detect a moderate or large (2.0<HR≤3.0) effect of the genetic risk exposure when the prevalence of the risk exposure was not very low (≥0.1) and the disease of interest was not rare (such as diabetes and dementia). The CLSA had enough power to detect a large effect of the gene-environment interaction only when both risk exposures had relatively high prevalence (0.2) and the disease of interest was very common (such as diabetes). The minimum detectable hazard ratios (MDHR) of the CLSA for the environmental and genetic risk exposures obtained from this simulation study were larger than those calculated according to the conventional sample size calculation method. For example, the MDHR for the environmental risk exposure was 1.15 according to the conventional method if the prevalence of the risk exposure was 0.1 and the disease of interest was dementia. In contrast, the MDHR was 1.61 if the same exposure was measured every 3 years with a misclassification rate of 0.1 according to this simulation study. With a given sample size, higher statistical power could be achieved by increasing the measuring frequency in participants with high risk of declining health status or changing risk exposures, and by increasing measurement accuracy of diseases and risk exposures. A properly designed simulation-based sample size calculation is superior to conventional methods when rigorous sample size calculation is necessary.

## Introduction

Biological and technological advances over the past decade, such as human genome sequencing, have increased researchers’ ability to study aging in all its complexity. The importance of studying gene-environment interactions in the context of aging related chronic diseases has been emphasized since they typically occurred as a result of the interaction between an individual’s genetic makeup and detrimental environmental circumstances [1]. To detect the effect of a genetic factor or a gene-environment interaction with a sufficient statistical power, the required sample size of a case-control study is expected to be extraordinary large and up to several hundred thousand [2]. Therefore, many genetic association studies were susceptible to lack of sufficient statistical power [3]. To date, very few longitudinal studies on aging have collected biomarker, genetic or epigenetic data to elucidate the process of aging and how biological processes interact with the physical and psychosocial environment to produce deleterious health outcomes.

Unlike early association studies in which individuals were not tracked over time and all measurements on each participant were made at a given point in time, a longitudinal study, which involves several observations of the same subjects over a period of time, enables researchers to separate the changes over time within subjects (i.e. aging effects) from differences between subjects at baseline (i.e. cohort effects). It also allows researchers to create the most comprehensive and insightful framework for understanding the mechanisms by which genome function can be altered during the process of aging [4, 5]. Moreover, genotyping costs have decreased dramatically over the last decade—making the use of the longitudinal design feasible to investigate the gene-environment interactions in diseases. However, longitudinal studies are time consuming, costly, and subject to high attrition rates. For longitudinal studies of gene-environment interactions in diseases, the sample size remains limited by the cost of proper phenotyping [6]. Therefore, rigorous sample size or statistical power estimation is crucial to ensure that such a study is scientifically beneficial and cost-effective.

The present simulation study is motivated by the design of the Canadian Longitudinal Study on Aging (CLSA), which is a national multi-disciplinary study investigating the complexities of the aging process, and seeking to improve the understanding of the transitions and trajectories of healthy aging [7]. The CLSA consists of a national stratified random sample of 50,000 Canadian women and men between the ages of 45 and 85 years at the time of recruitment (baseline). Participants will undergo repeated waves of data collection every three years and will be followed for at least twenty years, or until death. All participants will be asked to provide a common set of information on demographic, social, physical/clinical, psychological, economic, and health service utilization aspects relevant to health and aging. Of the 50,000 participants, 30,000 (the CLSA comprehensive cohort) will also be asked to provide additional in-depth information through physical examinations and biological specimen collection. The choice of data collection frequency, i.e. every three years, balances the need to have a short enough interval to capture important changes and map trajectories with the practical consideration of the time required to complete a wave of data collection. The inclusion of study participants as young as 45 years of age at baseline is motivated by the desire to capture mid-life experiences prospectively, since important changes known to influence outcomes later in life occur during this period. The lower age limit will also permit inclusion of individuals who are part of the baby boom cohort (i.e. those born between 1946 and 1964), who were 47 to 65 years of age in 2011. The upper limit includes individuals entering their senior years who are making the transition into retirement, who are already retired, and who have already reached old age. In the CLSA comprehensive cohort, self-reported diagnoses of chronic conditions are supplemented with a disease-specific questionnaire and physical exam.

Determinants of the required sample size for a longitudinal study include: (1) study objectives (to provide reliable sample size calculation, an appropriate statistical test for the hypotheses of interest, which should be established to reflect the study objectives, is necessarily derived under the study design); (2) type of endpoints/outcomes (continuous, binary, categorical, or survival); (3) variation of the study population; (4) type I error (probability of rejecting the null hypothesis when it is true) and type II error (probability of not rejecting the null hypothesis when it is false); (5) minimum clinically important effect size; (6) measurement errors in outcomes and risk exposures; (7) length of follow-up; (8) time and frequency of repeated measures; (9) correlation between repeated measures on the same subject; (10) attrition due to mortality and loss to follow-up; (11) sampling strategy; and (12) unmeasured etiological determinants, etc. To estimate the required sample size or statistical power of a population-based longitudinal study with time to a particular disease as the outcome, such as the CLSA, conventional sample size calculation methods using formulae [8, 9, 10] or software packages (such as STATA, SAS, and PASS) are available. However, these methods tend to overestimate the statistical power by overlooking some of the above determinants of the required sample size, especially the measurement errors, unmeasured etiological determinants, and competing events that can impede the occurrence of the event of interest. In contrast, a simulation-based sample size calculation, if designed properly, allows the above determinants of the required sample size to be taken into account simultaneously and offers flexibility in accommodating complex study design features and incorporating complicated statistical model which matches the underlying data.

The objectives of this simulation study are to: (1) investigate the statistical power profile of the CLSA to detect the effect of environmental and genetic risk factor, and their interaction on age-related chronic diseases, with the unmeasured etiological determinant, delayed entry into the study, errors in measuring risk exposures, frequency and time of the repeated measures, and the fact that the risk of developing an age-related chronic disease increases over time being taken into account; and (2) explore the design alternatives and implementation strategies for increasing the statistical power of population-based longitudinal studies in general; and (3) provide a practical example on how to conduct sample size and statistical power calculation using a simulation study.

## Materials and Methods

When participants of the CLSA enter into the study, they either sustain the age-related disease of interest or are free of that disease. During the course of the study, participants may die before ever developing that disease, develop the disease and then die, live with or without the disease until the end of the study. Since most age-related chronic diseases are not curable, we assume that participants will live with the disease until the end of their lives. To mimic the evolution of the CLSA cohort overtime, an irreversible illness-death model [11] was adopted as both the simulation and analytical models in this simulation study. Compared to other survival analysis models focusing on the transition from healthy to diseased stage only, the illness-death model allows the transition from healthy to dead stage be taken into account as the competing risk for the transition from healthy to diseased stage. In this section, we presented in detail the illness-death model, design of the simulation study, and how the increasing hazard of developing an age-related disease over time and delayed entry into the study (i.e. participants entered into the study at different ages) were incorporated into the simulation study.

### Irreversible illness-death model

The irreversible illness-death model [11] is widely used in medical literature to describe the progression of incurable diseases over time between three states: “healthy”, “diseased”, and “dead” (absorbing state). In this paper, the research interest lies in the transition from “healthy” to “diseased” while the transition from “healthy” to “dead” is considered as a competing risk.

Let 1, 2, and 3 denote “healthy”, “diseased”, and “dead” respectively, *t* denote the time since entering into a state, and *Z*(*t*) denote the state at time *t* for a subject. The transition intensity matrix is given by

$$Q(t|X)=\left(\begin{array}{ccc} -q_{12}(t|X)-q_{13}(t|X) & q_{12}(t|X) & q_{13}(t|X)\\ 0 & -q_{23}(t|X) & q_{23}(t|X)\\ 0 & 0 & 0 \end{array}\right),$$ (1)

where

$$q_{rs}(t|X)=\underset{\Delta t\to 0}{\text{lim}}P(Z(t+\Delta t)=s|Z(t)=r,\;X)/\Delta t$$ (2)

is the intensity of a transition from state *r* to state *s*, for *r*, *s* ∈ {1, 2, 3} and *s* ≠ *r*, and *X* represents a set of individual-level explanatory variables. Given time interval (*t*_1_, *t*_2_], the transition probability matrix is

$$P(t_{1},\;t_{2}|X)=\left(\begin{array}{ccc} p_{11}(t_{1},\;t_{2}|X) & p_{12}(t_{1},\;t_{2}|X) & 1-p_{11}(t_{1},\;t_{2}|X)-p_{12}(t_{1},\;t_{2}|X)\\ 0 & p_{22}(t_{1},\;t_{2}|X) & 1-p_{22}(t_{1},\;t_{2}|X)\\ 0 & 0 & 1 \end{array}\right)$$ (3)

where

$$p_{rs}(t_{1},\;t_{2}|X)=\text{Pr}(Z(t_{2})=s|Z(t_{1})=r,\;X)$$ (4)

is the transition probability from state *r* to state *s*, and

$$p_{11}(t_{1},\;t_{2}|X)=\text{exp}(-{\text{Q}}_{12}(t_{1},\;t_{2}|X)-{\text{Q}}_{13}(t_{1},\;t_{2}|X))$$ (5)

$$p_{22}(t_{1},\;t_{2}|X)=\text{exp}(-{\text{Q}}_{23}(t_{1},\;t_{2}|X))$$ (6)

$$p_{12}(t_{1},\;t_{2}|X)=\int_{t_{1}}^{t_{2}}p_{11}(t_{1},t|X)q_{12}(t|X)p_{22}(t,t_{2}|X)dt.$$ (7)

For 1≤*r*≤*s*≤3, the cumulative hazard function for transition from state *r* to state *s* is given by

$${\text{Q}}_{r\text{s}}(t_{1},\;t_{2}|X)=\int_{t_{1}}^{t_{2}}q_{\text{rs}}(t|X)dt.$$ (8)

### Weibull distribution with left truncation

The risk of developing an age-related chronic disease for a subject increases over time, which should be captured in the statistical analysis especially when the follow-up time is long. Therefore, the transition time between two given states is assumed to follow a Weibull distribution with shape parameter larger than one in this simulation study. In addition, the time when a subject initially comes under observation in a population-based cohort study usually does not coincide with the time when the subject becomes at risk of a disease, which implies the actual time a participant enters the study (baseline) may not be an appropriate time origin in survival analysis. Alternatively, a specific age, such as the lower bound 45 years in the CLSA, may be a reasonable choice of the time origin since the aging process, as conventionally believed, begins approximately at 45 years old. In this case, the survival time for a subject is defined as the elapsed time from 45 years old until the event of interest occurs or until the subject leaves the study, whichever occurs first; while the delayed entry into the study (entering the study after 45 years old) is considered as left-truncation occurring at the age of entry into the study [12].

Suppose a random variable *W*~Weibull(*λ*, *ρ*), where *λ* and *ρ* are the scale and shape parameters respectively. Its probability density function *f*_*W*_(⋅) and cumulative distribution function *F*_*W*_(⋅) are given by

$$f_{W}(w)=\rho /\lambda {\left(w/\lambda \right)}^{\rho -1}\text{exp}(-{\left(w/\lambda \right)}^{\rho }),$$ (9)

$$F_{W}(w)=1-\text{exp}(-{\left(w/\lambda \right)}^{\rho }).$$ (10)

Let *T* be a random variable obtained by left truncating *W* at 0≤*l*<∞. Its probability density function *f*_*T*_(⋅) and cumulative distribution function *F*_*T*_(⋅) are given by

$$f_{T}(t)=\{\begin{array}{cc} f_{W}(t)/(1-F_{W}(l)) & \text{if }t\geq l\\ 0 & \text{otherwise} \end{array}$$ (11)

$$F_{T}(t)=(F_{W}(t)-F_{W}(l))/(1-F_{W}(l)).$$ (12)

Both *W* and *T* have the same transition intensity:

$$q(w;\lambda ,\rho )=q(t;\lambda ,\rho ,l)=\rho /\lambda {\left(w/\lambda \right)}^{\rho -1}.$$ (13)

The survival time being left truncated at *l* can then be simulated from $t=F_{T}^{-1}(\mu )$, where *μ* is randomly sampled from a uniform(0,1) distribution.

### Simulation and analytical models

In this paper, both the simulation and analytical models were based on an irreversible illness-death model and implemented in the combination of SAS 9.2 (Cary, NC) and R 2.11 to achieve high computational efficiency.

Let $\lambda_{i}^{rs}$ and $\rho_{i}^{rs}$ be the Weibull scale and shape parameter for the transition from state *r* to state *s* for subject *i* with age of *l*_*i*_ + 45 at baseline, the transition time from “healthy” to “diseased” was generated by:

$$t_{i}^{12}=\lambda_{i}^{12}{\left(-\text{ln}(1-\mu_{i}(1-F_{W}(l_{i};\lambda_{i}^{12},\rho_{12}))+F_{W}(l_{i};\lambda_{i}^{12},\rho_{12}))\right)}^{1/\rho_{12}},$$ (14)

where

$$\lambda_{i}^{12}=\lambda_{i\;(0)}^{12}\text{exp}(\beta_{12}^{E}x_{i}^{E}+\beta_{12}^{G}x_{i}^{G}+\beta_{12}^{G\times E}x_{i}^{E}x_{i}^{G}+f_{i}),$$ (15)

$$\mu_{i}\sim \text{Uniform}(0,\;1),$$ (16)

$$f_{i}\sim \text{Normal}(0,\;0.585^{2}),$$ (17)

$\lambda_{i\;(0)}^{12}$ is the baseline scale parameter, which was carefully chosen to ensure the expected value of $\lambda_{i}^{12}$ is equal to the Weibull scale parameter estimated according to the incidence of the disease of interest for the Canadian population. $x_{i}^{G}$ and $x_{i}^{E}$ are genetic and environmental risk factors respectively. $\beta_{12}^{G}$, $\beta_{12}^{E}$ and $\beta_{12}^{G\times E}$ are the natural logarithm of hazard ratios for genetic risk factor, environmental risk factor and their interaction respectively. The transition time from “healthy” to “dead” was generated by:

$$t_{i}^{13}=\lambda_{i}^{13}{\left(-\text{ln}(1-\mu_{i}(1-F_{W}(l_{i};\lambda_{i}^{13},\rho_{13}))+F_{W}(l_{i};\lambda_{i}^{13},\rho_{13}))\right)}^{1/\rho_{13}},$$ (18)

where *μ*_*i*_~Uniform(0, 1), and $\lambda_{i\;}^{13}$ is the scale parameter estimated according to the mortality of the Canadian population. The time from entering into the study to loss to follow-up $t_{i\;}^{LTFU}$ for subject *i* was generated from $t_{i\;}^{LTFU}\sim \text{Exponential}(0.005)$.

When analyzing the simulated data, the scale parameter for transition from “healthy” to “diseased” was assumed to be

$$\lambda_{i}^{12}=\lambda_{i\;(0)}^{12}\text{exp}(\beta_{12}^{E}x_{i}^{E}+\beta_{12}^{G}x_{i}^{G}+\beta_{12}^{G\times E}x_{i}^{E}x_{i}^{G}).$$ (19)

The frailty term *f*_*i*_, which was incorporated in Eq (14) for simulating data but omitted in Eq (19) for analyzing data, represents the unmeasured etiological determinants. For subject *i*, if disease is observed at *t*_*i*_, the contribution of this individual to the likelihood is

$$f_{T}(t_{i})=f_{W}(t_{i})/(1-F_{W}(l_{i})).$$ (20)

If death or loss to follow-up is detected at *t*_*i*_, the data of this subject is considered as right censored (denoted by *C*) at *t*_*i*_. The contribution of this individual to the likelihood is

$$1-F_{T}(t_{i})=(F_{W}(t_{i})-F_{W}(l_{i}))/(1-F_{W}(l_{i})).$$ (21)

The likelihood to be maximized for all subjects is

$$L=\sum_{i=1}^{30000}\text{log}({\left(f_{W}(t_{i})/(1-F_{W}(l_{i}))\right)}^{1-C}{\left((F_{W}(t_{i})-F_{W}(l_{i}))/(1-F_{W}(l_{i}))\right)}^{C}).$$ (22)

### Choice of simulation parameters

Simulation parameters were carefully chosen to mimic the evolution of the CLSA comprehensive cohort. An instantaneous loss to follow-up rate of 0.005 per year was assumed (according to the information provided by Statistics Canada for the National Population Health Survey (NPHS) for the period 1994–1995 to 2000–2001). Consequently, 8% of participants will be lost to follow-up by the end of the CLSA. To incorporate this in the simulation, we assumed the time to loss to follow-up followed an exponential distribution with a rate parameter of 0.005. Both environmental and genetic risk factors were assumed to be dichotomous, which led to a more conservative statistical power in contrast to risk factors which were assumed to be continuous.

Choices of the prevalence of both risk factors were 0.01, 0.1, and 0.2 to represent very rare, common, and very common risk exposures respectively. To keep the simulation study simple yet representative, the statistical power profile of the CLSA for detecting three diseases was investigated. The diseases investigated were diabetes, dementia, and Parkinson’s disease, which represented very common, relatively common, and vary rare diseases in the study population respectively. These three diseases were considered in this simulation study as diseases with relatively quick, relatively slow, and very slow progression from “healthy” to “diseased” respectively. Therefore, 9 simulation scenarios were explored according to different combinations of the prevalence of risk exposure and the speed of progression from “healthy” to “diseased” state. We assumed the development of one disease as independent from the development of other diseases for each subject. Choices of the prevalence of diseases were 0.02 for dementia and Parkinson’s disease, and 0.14 for diabetes. Assuming 30,000 subjects were randomly sampled from the Canadian population between the ages of 45 to 85 years, the expected number of prevalent cases at baseline, and the expected number of deaths and incident cases at each year during the study period could be estimated based on the prevalence, incidence, and mortality of these diseases among Canadian adults. The Weibull scale and shape parameters for transitions between states were estimated by fitting a Weibull regression without adding covariates (R code was provided in S1 Text). For transition from “healthy” to “diseased”, the Weibull scale and shape parameters were 65 and 2.0 for diabetes, 48 and 5.6 for dementia, 130 and 3.3 for Parkinson’s disease. For transition from “healthy” to “dead”, the Weibull scale and shape parameters were fixed at 42 and 4.3. A log-normal frailty, modelled through a random effect with variance reflecting a 10-fold ratio in baseline risk between individuals on 97.5 and 2.5 population percentile, was assumed when simulating the data to represent the unmeasured etiological determinants.

Misclassification or measurement error in risk factors is typically thought to be non-differential in cohort studies since the exposure assessment is independent of the disease diagnosis, i.e. the probability of misclassification is the same among diseased and non-diseased subjects [13]. In this simulation study, choices of non-differential misclassification rate for environmental and genetic risk factors were 0.1 and 0.01 respectively. Repeated measures were assumed to be taken at baseline and every 3 years thereafter up to 21-year follow-up. When investigating the power profile for detecting the main effect of one risk exposure, the hazard ratios for another risk exposure and gene-environment interaction were set to 1. Similarly when investigating the power profile for detecting gene-environment interaction, hazard ratios for the main effects of both risk exposures were set to 1.5.

To achieve a reasonable degree of precision for estimating the statistical power, 1000 datasets for each scenario were simulated. Within each dataset, thirty thousand subjects were generated to represent the sample size of the CLSA comprehensive cohort. In this study, different significance levels were used for testing the null hypothesis for the environmental risk exposure, genetic risk exposure, and their interaction. For the environmental risk exposure, conventional significance level of 0.05 was used. For the genetic risk exposure and gene-environment interaction, both 10^−4^ and 5×10^−8^ were used, with 10^−4^ being considered as a threshold under the circumstance where the genetic exposure is defined on the basis of a variant lying in a vaguely defined candidate gene based on biological plausibility or linkage-based genomic positioning [14] and 5×10^−8^ being considered as an acceptable genome-wide significance threshold [15]. The statistical power was estimated as the proportion of simulated datasets in which the null hypothesis of no exposure effect was rejected at the above significant levels. The minimum detectable hazard ratio (MDHR) was defined as the smallest hazard ratio that could be detected with a sample size of 30,000 and power of 80% at the above level of statistical significance. It was categorized into four categories: small (1.0<MDHR≤1.5); moderate (1.5<MDHR≤2.0); large (2.0<MDHR≤3.0); and substantial (MDHR>3.0). The design of the simulation study was illustrated in detail in S2 Text.

## Results

### The number of prevalent and incident cases

**Fig 1:**
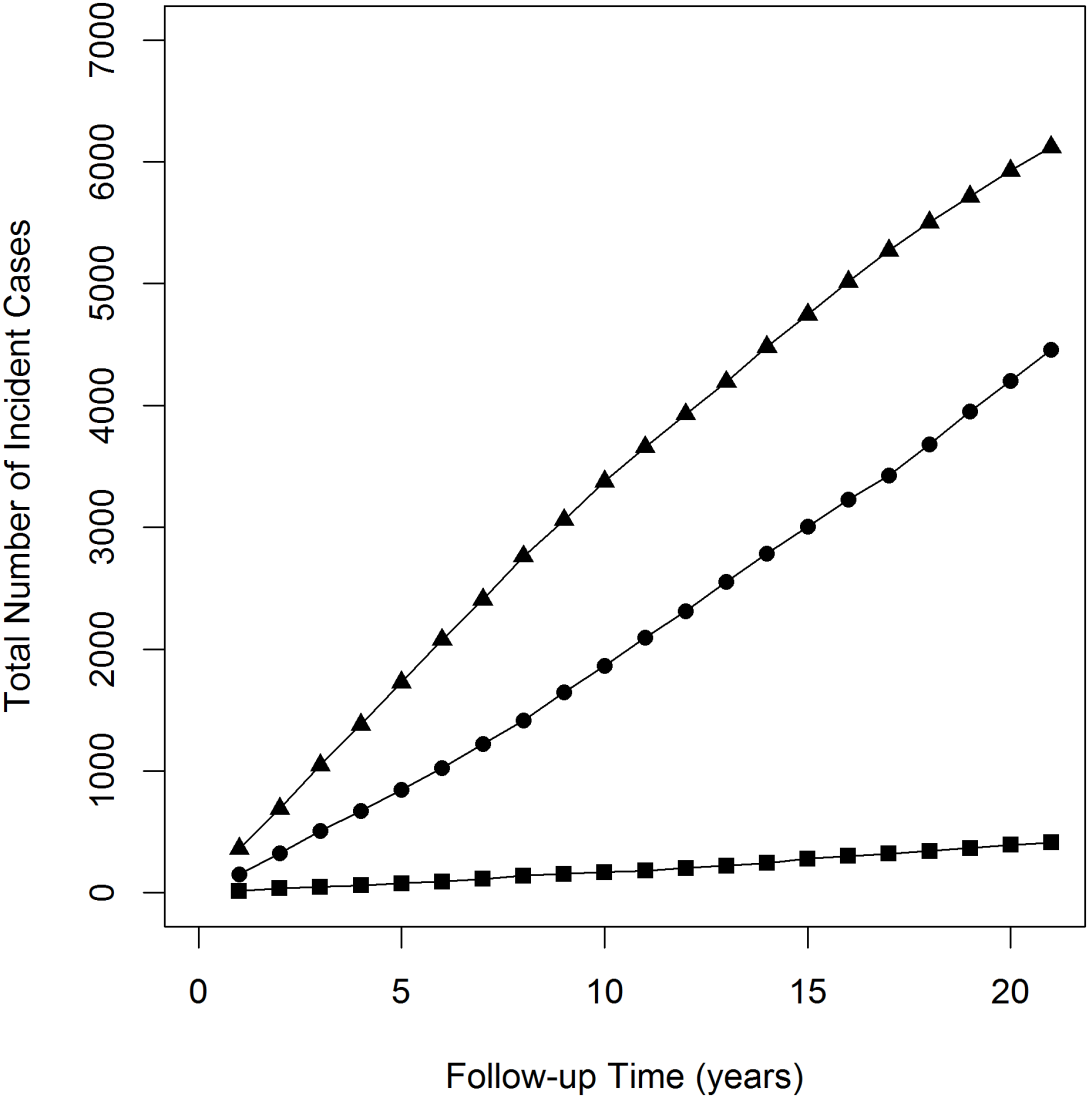
Total Number of Incident Cases in the Follow-up Period. Square, circle and triangle represent the total number of incident cases for Parkinson disease, dementia, and diabetes respectively.

Among the 30,000 subjects, the estimated number of subjects with diabetes, dementia and Parkinson’s disease at baseline were approximately 4000, 600 and 500 respectively. The total number of incident cases from baseline increased substantially for both diabetes and dementia over the follow-up period. After 21 years of follow-up, the total number of incident cases was approximately 6100 for diabetes and 4400 for dementia. In contrast, the total number of incident cases for Parkinson’s disease increased slowly and the total number of incident cases was approximately 420 after 21 years of follow-up. The estimated total number of incident cases over the follow-up period for each disease was presented in Fig 1.

### Statistical power profile of the CLSA

**Fig 2:**
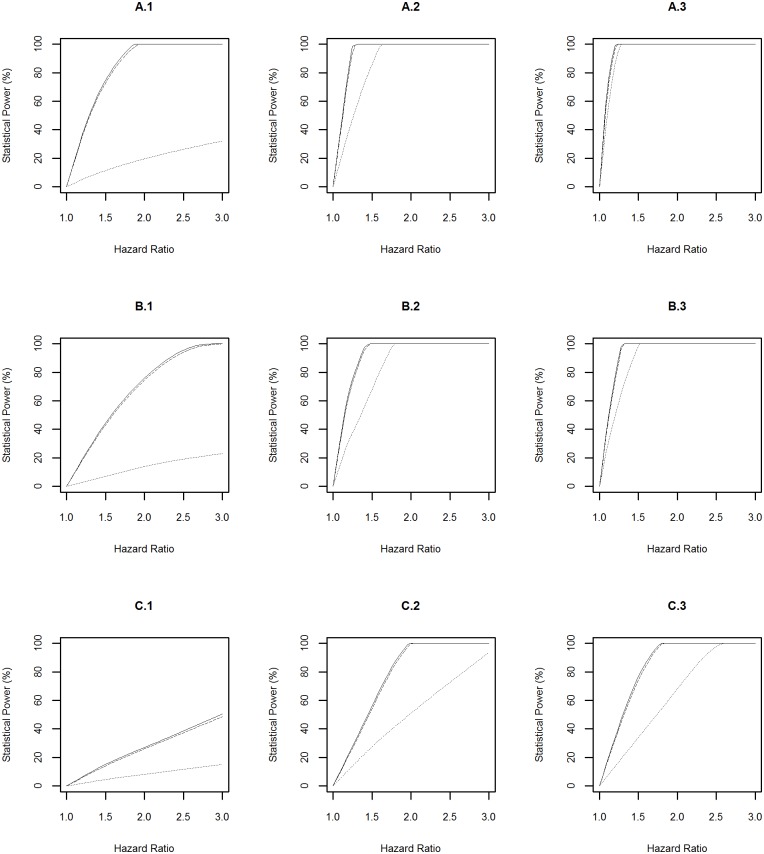
Power Profile at Significance Level of 0.05 for Environmental Risk Exposure. “A”, “B” and “C” represent diabetes, dementia and Parkinson’s disease respectively. “1”, “2” and “3” represent rare (prevalence = 0.01), common (prevalence = 0.1), and very common (prevalence = 0.2) environmental risk exposures respectively. The solid, dashed and dotted lines represent the statistical power profile of the study assuming subjects undergo health monitoring continuously, subjects undergo repeated measures every three years, and subjects undergo repeated measures every three years and the environmental risk exposure is measured with a misclassification rate of 0.1 respectively.

Statistical power profiles of the CLSA for assessing the association between environmental risk exposures and diseases at the significance level of 0.05 were presented in Fig 2. Among the 9 simulation scenarios, the highest statistical power was achieved to identify the association between a risk factor with prevalence of 0.2 and diabetes (A.3 in Fig 2). In this scenario, the power reached above 99% to detect a hazard ratio of 1.3 even when the risk exposure was measured with a misclassification rate of 0.1. In contrast, the smallest statistical power was achieved to identify the association between a risk factor with prevalence of 0.01 and the Parkinson’s disease (C.1 in Fig 2). In this scenario, the power was 50% to detect a hazard ratio of 3.0 even when the risk exposure was measured precisely (i.e. misclassification rate = 0).

**Fig 3:**
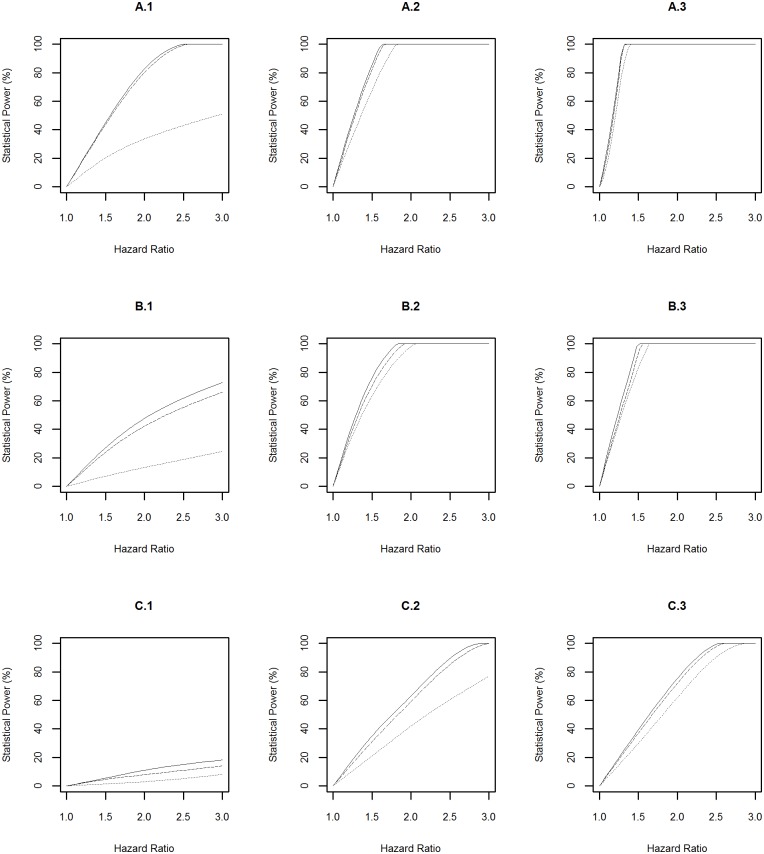
Power Profile at Significance Level of 0.0001 for Genetic Risk Exposure. “A”, “B” and “C” represent diabetes, dementia and Parkinson’s disease respectively. “1”, “2” and “3” represent rare (prevalence = 0.01), common (prevalence = 0.1), and very common (prevalence = 0.2) genetic risk factors respectively. The solid, dashed and dotted lines represent the statistical power profile of the study assuming subjects undergo health monitoring continuously, subjects undergo repeated measures every three years, and subjects undergo repeated measures every three years and the genetic risk exposure is measured with a misclassification rate of 0.1 respectively.

**Fig 4:**
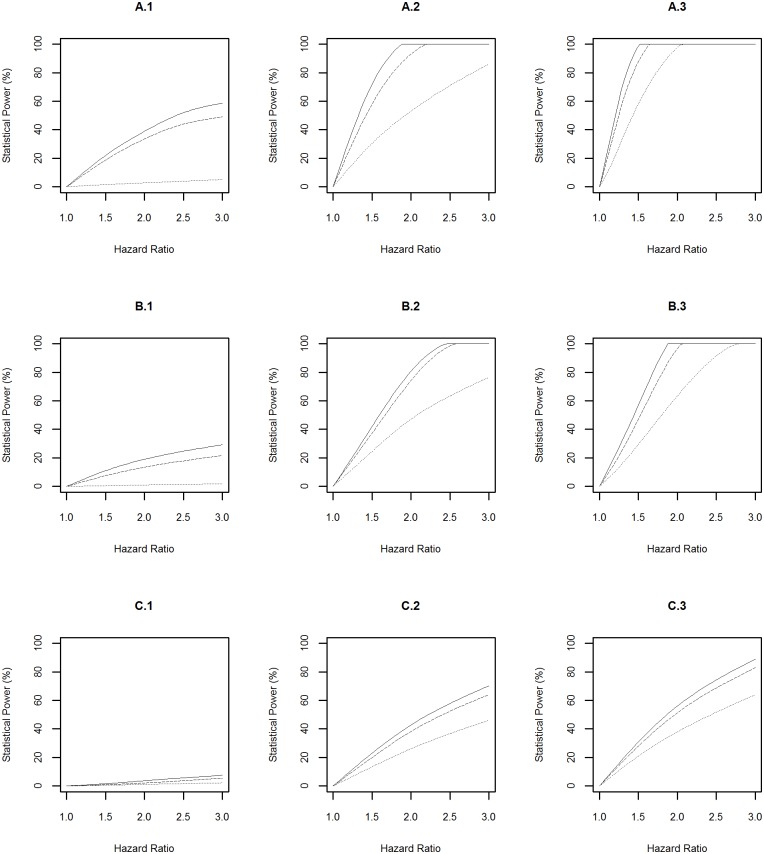
Power Profile at Significance Level of 5×10^−8^ for Genetic Risk Exposure. “A”, “B” and “C” represent diabetes, dementia and Parkinson’s disease respectively. “1”, “2” and “3” represent rare (prevalence = 0.01), common (prevalence = 0.1), and very common (prevalence = 0.2) genetic risk factors respectively. The solid, dashed and dotted lines represent the statistical power profile of the study assuming subjects undergo health monitoring continuously, subjects undergo repeated measures every three years, and subjects undergo repeated measures every three years and the genetic risk exposure is measured with a misclassification rate of 0.1 respectively.

The statistical power profiles for assessing the association between genetic risk exposures and diseases at the significance levels of 10^−4^ and 5×10^−8^ were presented in Figs 3 and 4 respectively. Similarly, the highest statistical power was achieved to identify the association between a risk factor with prevalence of 0.2 and diabetes (A.3 in Fig 3 and A.3 in Fig 4). In this scenario, the power reached above 99% to detect hazard ratios of 1.4 and 2.1 at the significance levels of 10^−4^ and 5×10^−8^ respectively when the risk exposure was measured with a misclassification rate of 0.01. The smallest statistical power was achieved to detect the association between a risk factor with prevalence of 0.01 and Parkinson’s disease (C.1 in Fig 3 and C.1 in Fig 4). In this scenario, the power was <20% to detect a hazard ratio of 3.0 at either significance level even when the risk exposure was measured accurately.

**Fig 5:**
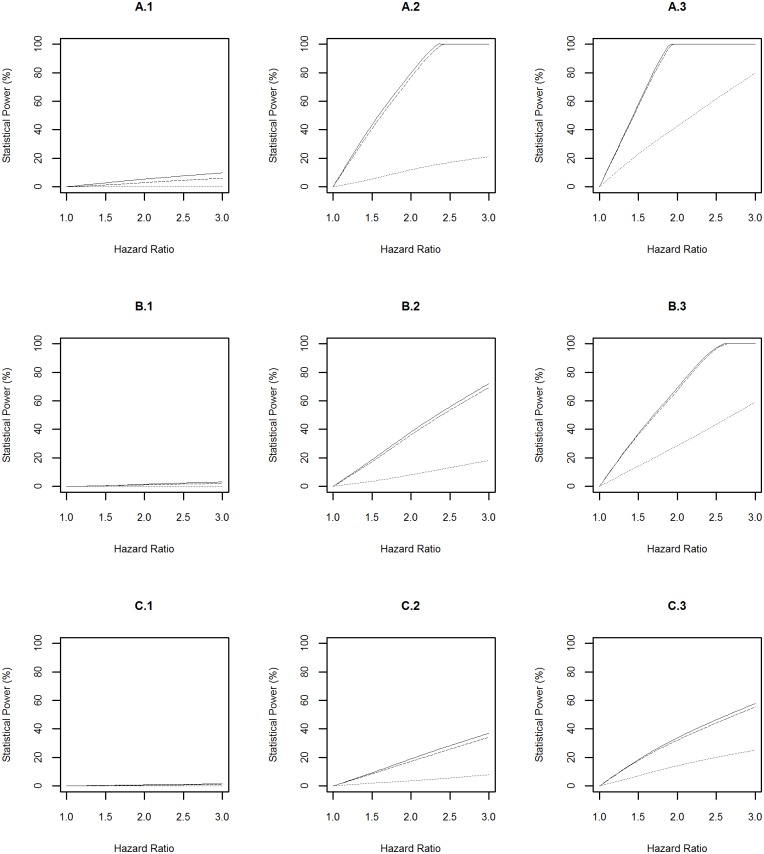
Power Profile at Significance Level of 0.0001 for Gene-Environment Interaction. “A”, “B” and “C” represent diabetes, dementia and Parkinson’s disease respectively. “1”, “2” and “3” represent that both environmental and genetic risk factors are rare (prevalence = 0.01), common (prevalence = 0.1), and very common (prevalence = 0.2) respectively. The solid, dashed and dotted lines represent the statistical power profile of the study assuming subjects undergo health monitoring continuously, subjects undergo repeated measures every three years, and subjects undergo repeated measures every three years and the both environmental and genetic risk exposures are subject to measurement error (0.1 for environmental risk exposure and 0.01 for genetic risk exposure) respectively.

**Fig 6:**
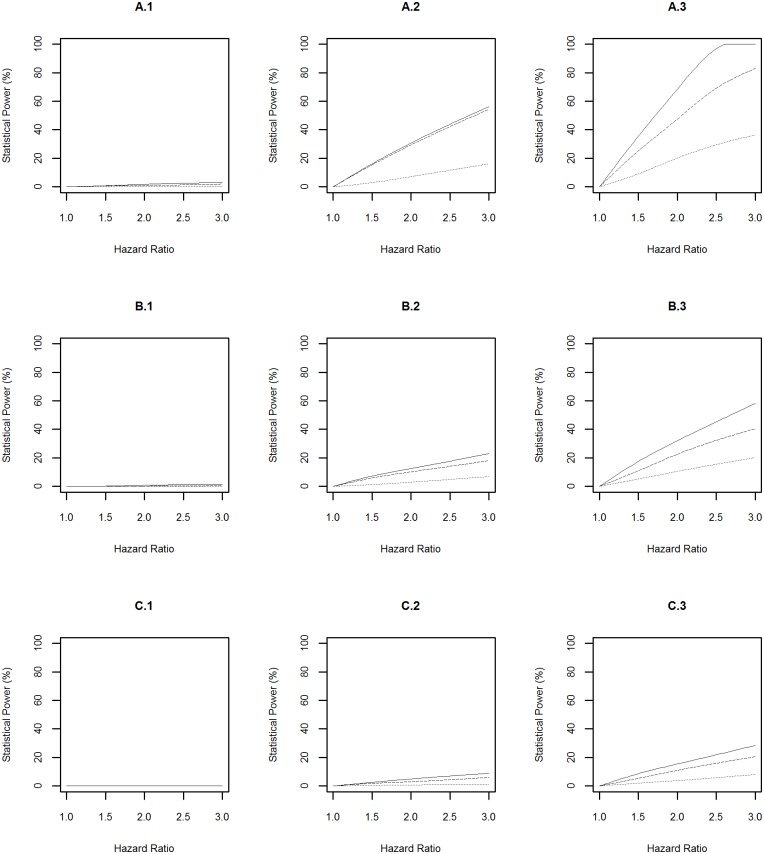
Power Profile at Significance Level of 5×10^−8^ for Gene-Environment Interaction. “A”, “B” and “C” represent diabetes, dementia and Parkinson’s disease respectively. “1”, “2” and “3” represent that both environmental and genetic risk factors are rare (prevalence = 0.01), common (prevalence = 0.1), and very common (prevalence = 0.2) respectively. The solid, dashed and dotted lines represent the statistical power profile of the study assuming subjects undergo health monitoring continuously, subjects undergo repeated measures every three years, and subjects undergo repeated measures every three years and the both environmental and genetic risk exposures are subject to measurement error (0.1 for environmental risk exposure and 0.01 for genetic risk exposure) respectively.

The statistical power profiles of the CLSA for assessing the association between gene-environment interactions and diseases at significance levels of 10^−4^ and 5×10^−8^ were presented in Figs 5 and 6 respectively. If the environmental and genetic risk exposures were measured with misclassification rates of 0.1 and 0.01 respectively, the statistical power was less than 80% to detect a hazard ratio of 3.0 at significance levels of 10^−4^ and 5×10^−8^ even when both environmental and genetic risk exposures had high prevalence (0.2) and the disease of interest was diabetes, a very common disease with fast progression from “healthy” to “diseased” state (A.3 in Fig 5 and A.3 in Fig 6).

The above power profiles also showed that the statistical power to identify the effect of environmental and genetic risk exposures, and their interaction on a disease was boosted when: (1) the prevalence of the risk exposures increased; (2) the disease of interest is common in the population, i.e. the progression from “healthy” to “diseased” was fast; (3) risk exposures were measured accurately (i.e. misclassification rate = 0). In addition, the frequency of data collection every three years led to a slightly lower statistical power compared to the design with assuming that participants underwent health monitoring continuously.

### Minimum detectable hazard ratio

The MDHR for the environmental risk exposures were presented in Table 1. For environmental risk exposures with a high prevalence (0.2), the CLSA had sufficient power to detect small hazard ratios (HR) (i.e. 1.0<MDHR≤1.5) even when the risk exposure was measured with a misclassification rate of 0.1 for any of the three diseases investigated in this simulation study, no matter the disease was common or rare. For risk exposures with a prevalence of 0.1, the CLSA had sufficient power to detect small, moderate HR (1.5<MDHR≤2.0) and large HR (2.0<MDHR≤3.0) if the disease of interest was diabetes, dementia, and Parkinson’s disease respectively when the risk exposure was measured with a misclassification rate of 0.1. For risk exposures with a very low prevalence (0.01), the CLSA had no enough power to detect even a large HR if the risk exposure was measured with a misclassification rate of 0.1.

**Table 1:** Minimum detectable hazard ratio for environmental risk exposure.

Characteristic of the risk exposure		Frequency of outcome measurement	Minimum detectable hazard ratio (α = 0.05)* Disease of interest		
Prevalence	Misclassification rate		Diabetes	Dementia	Parkinson’s disease
0.01	0	Continuous	1.57	2.12	>3.00
			1.43^&^	1.53^&^	3.90^&^
	0	Every 3 years	1.61	2.27	>3.00
	0.1	Every 3 years	>3.00	>3.00	>3.0
0.1	0	Continuous	1.21	1.27	1.72
			1.13^&^	1.15^&^	1.57^&^
	0	Every 3 years	1.22	1.29	1.74
	0.1	Every 3 years	1.46	1.61	2.68
0.2	0	Continuous	1.13	1.21	1.53
			1.08^&^	1.11^&^	1.41^&^
	0	Every 3 years	1.14	1.23	1.56
	0.1	Every 3 years	1.18	1.29	2.17

Note:

* Minimum detectable hazard ratio is defined as the smallest hazard ratio that can be detected with a statistical power of 80% at the significance level of 0.05.

^&^ The minimum detectable hazard ratio is obtained from conventional method for sample size calculation based on the proportional-hazard model proposed by Schoenfeld [Schoenfeld D. Sample-size formula for the proportional-hazards regression model. Biometrics 1983; 39: 499–503]

The MDHR for genetic risk exposures were presented in Table 2. At the significance level of 10^−4^, the CLSA had sufficient power to detect small HR for diabetes and dementia but a large HR for Parkinson’s disease if the prevalence of the risk exposure was 0.2, and to detect a moderate HR for diabetes and dementia if prevalence of the risk exposure was 0.1, provided that the risk exposure was subject to measurement error (misclassification rate = 0.01). When the prevalence of the risk exposure was 0.01, the CLSA had sufficient power to detect moderate HR for diabetes only if the risk exposure was measured accurately. At the significance level of 5×10^−8^, the CLSA had sufficient power to detect moderate and large HR for diabetes and dementia only if the risk exposure was measured with a misclassification rate of 0.01. When the prevalence of the risk exposure was 0.1, the CLSA had sufficient power to detect moderate HR for diabetes and large HR for dementia if the risk exposure was measured accurately. When the prevalence of the risk exposure was 0.01, the CLSA did not have sufficient power to detect even a large HR for any diseases.

**Table 2:** Minimum detectable hazard ratio for genetic risk exposure.

Characteristic of the risk exposure		Frequency of outcome measurement	Minimum detectable hazard ratio (α = 10^−4^)* Disease of interest			Minimum detectable hazard ratio(α = 5×10^−8^)* Disease of interest		
Prevalence	Misclassification rate		Diabetes	Dementia	Parkinson’s disease	Diabetes	Dementia	Parkinson’s disease
0.01	0	Continuous	1.95	>3.00	>3.00	>3.00	>3.00	>3.00
			1.84^&^	2.05^&^	9.97^&^	2.24^&^	2.59^&^	21.76^&^
	0	Every 3 years	2.00	>3.00	>3.00	>3.00	>3.00	>3.00
	0.01	Every 3 years	>3.00	>3.00	>3.00	>3.00	>3.00	>3.00
0.1	0	Continuous	1.46	1.55	2.31	1.58	1.98	>3.00
			1.22^&^	1.27^&^	2.16^&^	1.31^&^	1.37^&^	2.77^&^
	0	Every 3 years	1.50	1.61	2.39	1.75	2.10	>3.00
	0.01	Every 3 years	1.62	1.71	>3.00	2.75	>3.0	>3.0
0.2	0	Continuous	1.24	1.39	2.08	1.34	1.69	2.59
			1.16^&^	1.19^&^	1.79^&^	1.22^&^	1.27^&^	2.16^&^
	0	Every 3 years	1.24	1.43	2.15	1.45	1.81	2.86
	0.01	Every 3 years	1.25	1.48	2.34	1.73	2.28	>3.00

Note:

* Minimum detectable hazard ratio is defined as the smallest hazard ratio that can be detected with a statistical power of 80% at the significance levels of 10^−4^ and 5×10^−8^.

^&^ The minimum detectable hazard ratio is obtained from conventional method for sample size calculation based on the proportional-hazard model proposed by Schoenfeld [Schoenfeld D. Sample-size formula for the proportional-hazards regression model. Biometrics 1983; 39: 499–503]

The MDHR for gene-environment interactions were presented in Table 3. At the significance level of 10^−4^, the CLSA had sufficient power to detect moderate HR for diabetes, large HR for dementia and Parkinson’s disease, only if both risk exposures had high prevalence (0.2) and were measured accurately. At the significance level of 5×10^−8^, the CLSA had no sufficient power to detect even a large HR, even when the prevalence of both risk exposures was 0.2 and the disease of interest was very common, such as diabetes.

**Table 3:** Minimum detectable hazard ratio for gene-environment interaction.

Characteristic of risk exposures		Frequency of outcome measurement	Minimum detectable hazard ratio (α = 10^−4^)* Disease of interest			Minimum detectable hazard ratio(α = 5×10^−8^)* Disease of interest		
Prevalence	Misclassification rate		Diabetes	Dementia	Parkinson’s disease	Diabetes	Dementia	Parkinson’s disease
0.01^+^	0	Continuous	>3.00	>3.00	>3.00	>3.00	>3.00	>3.00
	0	Every 3 years	>3.00	>3.00	>3.00	>3.00	>3.00	>3.00
	0.1, 0.01^#^	Every 3 years	>3.00	>3.00	>3.00	>3.00	>3.00	>3.00
0.1^+^	0	Continuous	1.85	>3.00	>3.00	>3.00	>3.00	>3.00
	0	Every 3 years	2.00	>3.00	>3.00	>3.00	>3.00	>3.00
	0.1, 0.01^#^	Every 3 years	>3.00	>3.00	>3.00	>3.00	>3.00	>3.00
0.2^+^	0	Continuous	1.67	2.17	2.60	2.21	>3.00	>3.00
	0	Every 3 years	1.68	2.19	2.62	2.79	>3.00	>3.00
	0.1, 0.01^#^	Every 3 years	3.00	>3.00	>3.00	>3.00	>3.00	>3.00

Note:

* Minimum detectable hazard ratio is defined as the smallest hazard ratio that can be detected with a statistical power of 80% at the significance levels of 10^−4^ and 5×10^−8^.

^+^ Both environmental and genetic risk factor have the same specified prevalence.

^#^ Misclassification rate for environmental and genetic risk exposures are 0.1 and 0.01 respectively.

### Comparison of Minimum Detectable Hazard Ratio from Simulation and Conventional Method

The conventional method proposed by Schoenfeld [10] was commonly used in practice to calculate the required sample size or statistical power of a longitudinal study with survival outcome. However, this method assumed participants underwent health monitoring continuously and ignored the frequency and time of repeated measures and the unmeasured etiological determinants. The MDHR obtained using this method for the environmental and genetic risk exposures were presented in Tables 1 and 2 respectively. The MDHR calculated according to this method was smaller compared to that obtained from the simulation study under each scenario investigated in the present study. For example, the MDHR for the environmental risk exposure was 1.15 according to this conventional method when the prevalence of the environmental risk exposure was 0.1 and the disease of interest was dementia. In contrast, the MDHR for the same risk exposure and disease according to the present simulation study were 1.27 under the assumption that participants underwent health monitoring continuously, 1.29 under the assumption that participants underwent repeated measures every three years, and 1.61 under the assumption that participants underwent repeated measures every three years and the environmental risk exposure was subject to measurement error (misclassification rate = 0.1).

## Discussion

We found that the CLSA had enough power to detect the effect of any environmental risk factors and the effect of common genetic risk factors (prevalence≥0.1) for all diseases of interest. It was also capable of detecting the gene-environment interaction effect for diseases with fast or relatively slow progression from “healthy” to “diseased” when prevalence of both risk factors were high (≥0.2). However, the CLSA did not have enough power to detect the effect of gene-environment interaction for any disease of interest when the prevalence of the risk factors was not high (≤0.1).

We found the CLSA had sufficient power to detect a small or moderate effect of the environmental risk exposure, as long as the risk exposure and the disease of interest were not rare. It had enough power to detect a moderate or large effect of the genetic risk exposure when the prevalence of the risk exposure was not very low (≥0.1) and the disease of interest was not rare (such as diabetes and dementia). The CLSA had enough power to detect a large effect of the gene-environment interaction only when both risk exposures had relatively high prevalence (0.2) and the disease of interest was very common (such as diabetes).

Our results also showed the design of the CLSA with repeated measures every three years was a reasonable choice of frequency and time for data collection. This particular design allowed for substantial cost reduction while maintaining similar MDHR and statistical power in comparison to the design with exact event time being observed. This is because the frequency of the repeated measures, every three years, is considerably smaller than the mean sojourn in the “healthy” and “diseased” states, which ensures the transition from “healthy” to “diseased” and “diseased” to “dead” be observed with high probability. Enlightened by this finding, we believe, though not demonstrated in this simulation study, that if risk factors are also time-dependent, the frequency of repeated measures should be determined according to prior knowledge about the trajectories of changes in both outcomes and risk factors to achieve a higher statistical power with a fixed sample size. Additionally, recruiting a higher proportion of subjects who are believed to be experiencing or going to experience major health changes, or measuring these subjects more frequently may also be used as strategies for increasing the statistical power with an overall fixed sample size. For example, if subjects within a given age range are believed to be more likely to develop a disease of interest, including more subjects within that age range in the study sample may help to increase the statistical power of the study.

We illustrated that the frequency and time of the CLSA may be a reasonable design choice, however, it may not be optimal. Our results implied that slightly larger statistical power can be achieved by increasing the frequency of the repeated measures. This is consistent with the findings from the study by van den Hout *et al* [16], in which they illustrated that the length of follow-up and sample size of a study were associated with the performance of the estimation of life expectancies. Based on the simulation and analysis using a reversible illness-death model, they concluded that it was not always necessary to have a long follow-up or a large sample size and relatively short follow-up time could still be used if the time interval between measurements was not too wide. For the reversible illness-death model, which is used to model the progression of reversible diseases, a large amount of events or transitions may be observed through relatively small sample size but frequent measurement. This is due to the fact that a subject may experience several transitions from “healthy” to “diseased” and “diseased” to “healthy” during the follow-up period of the study. However, for the irreversible illness-death model used in this simulation study, which is more appropriate to model the progression of irreversible diseases, increasing the frequency of the measurements may only lead to a very slight increase in the number of observed events and the statistical power. In fact, three factors—the length of follow-up (i.e. the duration of the study), frequency of repeated measures, and sample size, can be adjusted to an optimal level to achieve higher statistical power with lower associated costs. Increasing frequency or duration may only increase power to an upper limit that depends on the progression of diseases for the study population, whereas increasing sample size can raise power toward 1.0. In general, increasing the sample size leads to an increase in the cost of recruitment; increasing the length of follow-up increases the risk of attrition and the cost of tracking participants. When assessments are expensive, increasing the frequency of repeated measures will, consequently, lead to increased expenses. Our further investigation will involve optimal designs with costs being taken into account.

In this simulation study, we found that misclassification of the environmental and genetic risk exposures substantially increased the MDHR and decreased the statistical power. This is consistent with the finding from Garcia-Closas *et al* [17], that misclassification of environmental or genetic risk factors can substantially increase the sample size required to evaluate gene-environment interaction in case-control studies. Therefore, improving the accuracy of measurement for both genetic and environmental risk exposures is critical, especially for a valid assessment of gene-environment interaction. Moreover, our results showed the effect of misclassification seemed not differential for diseases with slow or fast progression. This is because we only considered the misclassification of risk factors while assuming an accurate diagnosis of disease in this simulation study. Based on the results from this simulation study, we can reasonably infer that the misclassification in disease measurements will decrease the statistical power and the impact of misclassification will be larger for diseases with slow progression from “healthy” to “diseased” in comparison to those with fast progression.

We showed that the statistical power based on a proportional-hazards model substantially overestimated the statistical power due to overlooking important determinants. This suggested that a properly designed simulation-based sample size and statistical power estimation method should be used when rigorous sample size calculation is necessary.

This simulation study was designed based on certain assumptions, such as constant loss to follow-up rate over time, time-independent risk exposures, and accurate disease diagnosis. However, enlightened by the design and results of this simulation study, researchers will have a good understanding of the trend of the statistical power when these assumptions do not hold. To the best of our knowledge, this project is the first attempt to investigate the power profile of a population-based longitudinal study through a simulation study. This research provided a more realistic power profile by taking into account the measurement error, unmeasured etiological determinants, and competing events that can impede the occurrence of the event of interest, which are usually ignored by traditional sample size and statistical power calculation.

## Conclusions

A properly designed simulation-based sample size calculation method should be adopted when rigorous sample size calculation is necessary. Improving the design and implementation of a longitudinal study can increase its statistical power for detecting the effect of environmental and genetic risk exposures, and their interaction on chronic diseases. Moreover, the rationale and design of this simulation can be used as a practical example for estimating the required sample size for future longitudinal studies.

## Supporting Information

S1 Text Determine Weibull shape and scale parameter based on progression from healthy to diseased. (DOC)

S2 Text Design of the simulation study. (DOC)
