# Supplementary material for: Power Analysis for Population-Based Longitudinal Studies Investigating Gene-Environment Interactions in Chronic Diseases: A Simulation Study
Source: PLoS One. 2016 Feb 22;11(2):e0149940. doi: 10.1371/journal.pone.0149940 (PMC4762766; doi:10.1371/journal.pone.0149940)
Supplement: S1 Text — (DOC) [file pone.0149940.s001.doc]

**S1 Text. Resources and procedures for determining Weibull parameters**

**Table 1. Approximate distribution of the 30,000 CLSA comprehensive cohort by age and sex**

| **Age Band**  **(at baseline)** | **Approximate number of male subjects in CLSA**  **at Baseline** | **Approximate number of female subjects in CLSA**  **at Baseline** | **Approximate number of subjects in CLSA**  **at Baseline** | **Proportion of subjects among the 30,000 cohort** |
| --- | --- | --- | --- | --- |
| 45-49 | 3204 | 3216 | 6420 | 0.21 |
| 50-54 | 2793 | 2850 | 5643 | 0.19 |
| 55-59 | 2439 | 2493 | 4932 | 0.16 |
| 60-64 | 1824 | 1890 | 3714 | 0.12 |
| 65-69 | 1407 | 1518 | 2925 | 0.10 |
| 70-74 | 1197 | 1359 | 2556 | 0.09 |
| 75-79 | 927 | 1194 | 2121 | 0.07 |
| 80-84 | 594 | 939 | 1533 | 0.05 |
| 85 | 51 | 105 | 156 | 0.01 |

Resources: Estimated according to the age-sex break down for Canadians at the 2005 Census

**Table 2. Annual incidence of dementia and Parkinson’s disease (‰)**

| **Disease** | **Age Group** | | | | | | | | |
| --- | --- | --- | --- | --- | --- | --- | --- | --- | --- |
|  | **45-49** | **50-54** | **55-59** | **60-64** | **65-69** | **70-74** | **75-79** | **80-84** | **85+** |
| **Incidence** | | | | | | | | | |
| Dementia (male) ^1^ | 0 | 0 | 0 | 0 | 3.7 | 14.4 | 24.5 | 32.6 | 70.7 |
| Dementia (female) ^1^ | 0 | 0 | 0 | 0 | 6.8 | 7.6 | 17.7 | 36.7 | 69.5 |
| Parkinson (male) ^2^ | 0.11 | 0.11 | 0.11 | 0.54 | 0.54 | 1.33 | 1.33 | 2.13 | 2.13 |
| Parkinson (Female) ^2^ | 0.11 | 0.11 | 0.11 | 0.54 | 0.54 | 1.33 | 1.33 | 2.13 | 2.13 |
| Diabetes (male) ^3^ | 0.82 | 1.16 | 1.55 | 1.93 | 2.26 | 2.34 | 2.27 | 2.09 | 1.62 |
| Diabetes (female) ^3^ | 0.58 | 0.83 | 1.12 | 1.41 | 1.67 | 1.78 | 1.79 | 1.72 | 1.36 |
| **Prevalence** | | | | | | | | | |
| Dementia (male) ^4^ | 0 | 0 | 0 | 0 | 24 | 24 | 111 | 111 | 345 |
| Dementia (female) ^4^ | 0 | 0 | 0 | 0 | 24 | 24 | 111 | 111 | 345 |
| Parkinson (male) ^5^ | 0 | 0 | 16.39 | 15.9 | 15.9 | 39.51 | 39.51 | 90.91 | 90.91 |
| Parkinson (Female) ^5^ | 0 | 0 | 16.39 | 15.9 | 15.9 | 39.51 | 39.51 | 90.91 | 90.91 |
| Diabetes (male) ^3^ | 6.2 | 9.5 | 14.0 | 19.1 | 23.7 | 27.1 | 28.5 | 27.8 | 23.2 |
| Diabetes (female) ^3^ | 5.1 | 7.4 | 10.7 | 14.2 | 17.8 | 21.3 | 23.1 | 23.4 | 19.9 |

Sources:

1. Canadian Study of Health and Aging Working Group. The incidence of dementia in Canada. Neurology 2000; 55:66-73.
2. Morens DM, Davis JM, Grandinetti A, et al. Epidemiologic observations on Parkinson's disease: incidence and mortality in a prospective study of middle aged men. *Neurology* 1996;46:1044-50.
3. http://www.phac-aspc.gc.ca/cd-mc/publications/diabetes-diabete/facts-figures-faits-chiffres-2011/chap1-eng.php#Pre
4. Lindsay J, Sykes E, McDowell I, Verreault R, Laurin D. More than the epidemiology of Alzheimer’s Disease: contributions of the Canadian Study of Health and Aging. Can J Psychiatry 2004;49(2):83-91.
5. BioBasics, Government of Canada <http://www.biobasics.gc.ca/english/View.asp?x=771>

**Table 3. Estimated Weibull parameters for transition from Healthy to Diseased or Dead**

| Transitions | Scale parameter | Shape parameter |
| --- | --- | --- |
| Healthy to Dementia | 48 | 5.6 |
| Healthy to Parkinson | 130 | 3.3 |
| Healthy to Diabetes | 65 | 2.0 |
| Health to Dead | 42 | 4.3 |

R code for estimating Weibull shape and scale parameters

set.seed(5)

### generate data to mimic patients’ transition from health to diseased ###

gen_disease_data=function(para)

{

### age-gender Prevalence and incidence of diseases ###

### para==1 Dementia ###

### para==2 Parkinson ###

### para==3 Diabetes ###

if(para==1)

{

prev_male<-c(0,0,0,0,24,24,111,111,345)/1000

prev_female<-c(0,0,0,0,24,24,111,111,345)/1000

inci_male<-c(0,0,0,0,3.7,14.4,24.5,32.6,70.7,70.7)/1000

inci_female<-c(0,0,0,0,6.8,7.6,17.7,36.7,69.5,69.5)/1000

}

if(para==2)

{

prev_male<-c(0,0,16.39,15.9,15.9,39.51,39.51,90.91,90.91)/1000

prev_female<-c(0,0,16.39,15.9,15.9,39.51,39.51,90.91,90.91)/1000

inci_male<-c(0.11,0.11,0.11,0.54,0.54,1.33,1.33,2.13,2.13,2.13)/1000

inci_female<-c(0.11,0.11,0.11,0.54,0.54,1.33,1.33,2.13,2.13,2.13)/1000

}

if(para==3)

{

prev_male<-c(6.2,9.5,14.0,19.1,23.7,27.1,28.5,27.8,23.2,23.2)/100

prev_female<-c(5.1,7.4,10.7,14.2,17.8,21.3,23.1,23.4,19.9,19.9)/100

inci_male<-c(8.2,11.6,15.5,19.3,22.6,23.4,22.7,20.9,16.2,16.2)/1000

inci_female<-c(5.8,8.3,11.2,14.1,16.7,17.8,17.9,17.2,13.6,13.6)/1000

}

### age-gender mortality rate ###

death_male<-c(2.7,4.2,6.7,10.8,17.3,28.1,46,77.3,131.3,226.2)/1000

death_female<-c(1.8,2.7,4.1,6.8,10.4,17.4,29.4,51.4,93.6,191.6)/1000

### subject ID ###

pid<-c(1:30000)

### gender of subjects ###

sex<-c(rep(0,3204), rep(1,3216), rep(0,2793), rep(1,2850), rep(0,2439), rep(1,2493), rep(0,1824), rep(1,1890), rep(0,1407), rep(1,1518), rep(0,1197), rep(1,1359), rep

(0,927), rep(1,1194), rep(0,594), rep(1,939), rep(0,51), rep(1,105))

### age and age-group of subjects at baseline ###

age_group<-c(rep(1,3204), rep(1,3216), rep(2,2793), rep(2,2850), rep(3,2439), rep(3,2493), rep(4,1824), rep(4,1890), rep(5,1407), rep(5,1518), rep(6,1197), rep(6,1359),

rep(7,927), rep(7,1194), rep(8,594), rep(8,939), rep(9,51), rep(9,105))

age_base<-c(rep((45:49), 1284), rep((50:54), 1128), c(50,51,52), rep((55:59),986), c(55,56), rep((60:64),742), c(60,61,62,63), rep((65:69), 585), rep((70:74), 511), c

(70), rep((75:79), 424), c(75), rep((80:84), 306), c(80,81,82), rep(85,156))

### whether subjects are diseased at baseline ###

base_disease<-c(rbinom(3204,1,prev_male[1]), rbinom(3216,1,prev_female[1]), rbinom(2793,1,prev_male[2]), rbinom(2850,1,prev_female[2]), rbinom(2439,1,prev_male[3]),

rbinom(2493,1,prev_female[3]), rbinom(1824,1,prev_male[4]), rbinom(1890,1,prev_female[4]), rbinom(1407,1,prev_male[5]), rbinom(1518,1,prev_female[5]), rbinom(1197,1,prev_male

[6]), rbinom(1359,1,prev_female[6]), rbinom(927,1,prev_male[7]), rbinom(1194,1,prev_female[7]), rbinom(594,1,prev_male[8]), rbinom(939,1,prev_female[8]), rbinom(51,1,prev_male

[9]), rbinom(105,1,prev_female[9]))

### whether subjects are dead and time of death ###

dead<-rep(0, 30000)

time_dead<-rep(0, 30000)

### whether subjects are diseased and time when diseased during the follow-up period ###

time_disease<-rep(0, 30000)

disease<-base_disease

age<-age_base

for(year in 1:21)

{

age<-age+1

age_group<-as.integer((age-45)/5+1)

for(i in 1:30000)

{

if (age_group[i]>10)

{

age_group[i]=10

}

if (dead[i]==0)

{

if(sex[i]==0)

{

dead[i]<-rbinom(1,1,death_male[age_group[i]])

}

else

{

dead[i]<-rbinom(1,1,death_female[age_group[i]])

}

if (dead[i]==1)

{

time_dead[i]<-year

}

}

if(dead[i]!=1 && disease[i]!=1)

{

if(sex[i]==0)

{

disease[i]<-rbinom(1,1,inci_male[age_group[i]])

}

else

{

disease[i]<-rbinom(1,1,inci_female[age_group[i]])

}

if (disease[i]==1)

{

time_disease[i]<-year

}

}

}

}

for(i in 1:30000)

{

if(disease[i]==0 && dead[i]==1)

{

time_disease[i]=time_dead[i] ## if dead, time of diseased censored at time of dead ##

}

if(disease[i]==0 && dead[i]==0)

{

time_disease[i]=21 ## if not dead, time of diseased censored at the end of study ##

}

if(dead[i]==0)

{

time_dead[i]=21 ## if not dead, time of dead censored at the end of study ##

}

}

data<-data.frame(cbind(pid,age_base,sex, base_disease, disease,time_disease,dead, time_dead))

if(para==1)

{

write.table(data,"c:/dementia.txt", sep=" ", row.names=F)

}

else if(para==2)

{

write.table(data,"c:/parkinson.txt", sep=" ", row.names=F)

}

else if(para==3)

{

write.table(data,"c:/diabetes.txt", sep=" ", row.names=F)

}

}

gen_disease_data(1)

gen_disease_data(2)

gen_disease_data(3)

### estimate Weibull shape and scale parameters for transition ###

### from Healthy to Diseased and Health to Dead ###

library(survival)

est_shape_scale=function(para)

{

if(para==1)

{

data<-read.table("c:/dementia.txt", header=T, sep=" ")

}

else if(para==2)

{

data<-read.table("c:/parkinson.txt", header=T, sep=" ")

}

else if(para==3)

{

data<-read.table("c:/diabetes.txt", header=T, sep=" ")

}

healthy<-data[data$base_disease==0, ]

time_healthy_diseased<-healthy$time_disease+healthy$age_base-45

status_healthy_diseased<-healthy$disease

reg_healthy_diseased<-survreg(Surv(time_healthy_diseased,status_healthy_diseased)~1, dist="weibull")

healthy_diseased_shape<-1/reg_healthy_diseased$scale

healthy_diseased_scale<-exp(coef(reg_healthy_diseased))

time_healthy_dead<-healthy$time_dead+healthy$age_base-45

status_healthy_dead<-healthy$dead

reg_healthy_dead<-survreg(Surv(time_healthy_dead,status_healthy_dead)~1, dist="weibull")

healthy_dead_shape<-1/reg_healthy_dead$scale

healthy_dead_scale<-exp(coef(reg_healthy_dead))

time_dead<-data$time_dead+data$age_base-45

status_dead<-data$dead

reg_dead<-survreg(Surv(time_dead,status_dead)~1, dist="weibull")

dead_shape<-1/reg_dead$scale

dead_scale<-exp(coef(reg_dead))

return(data.frame(cbind(healthy_diseased_shape, healthy_diseased_scale, healthy_dead_shape, healthy_dead_scale, dead_shape, dead_scale)))

}

est_shape_scale(1)

est_shape_scale(2)

est_shape_scale(3)
