# Supplementary material for: Power Analysis for Population-Based Longitudinal Studies Investigating Gene-Environment Interactions in Chronic Diseases: A Simulation Study
Source: PLoS One. 2016 Feb 22;11(2):e0149940. doi: 10.1371/journal.pone.0149940 (PMC4762766; doi:10.1371/journal.pone.0149940)
Supplement: S2 Text — (DOC) [file pone.0149940.s002.doc]

**S2 Text. Design of the Simulation Study**

**A. Generate Baseline Data**

**Varied Parameters**

1.Prevalence of disease ($\pi_{D}$=0.02, 0.14)

2. Probability of genotype risk factor ($\pi_{G}$=0.01, 0.1, 0.2) and environmental risk exposure ($\pi_{E}$=0.01, 0.1,

0.2)

**Fixed Parameters**

1.Sample size (n=30,000)

2. Age-sex distribution for Canadian population aged 45-85

3. Misclassification rate for environmental and genotype risk factors are $M_{E}=0.1$ and $M_{G}=0.01$

respectively.

**Simulation procedure**

1.Generate age ($x_{A}$) and gender according age-sex breakdown for Canadian population age

from 45 to 85

2. Generate true genotype risk factor: $x_{G}\sim\text{Bernoulli(}\pi_{G}\text{)}$

3. Generate true environmental risk factor: $x_{E}\sim\text{Bernoulli(}\pi_{\text{E}}\text{)}$

4. Generate genotype risk factor with misclassification: $x_{G}^{O}=\left\{\begin{matrix} x_{G} & \text{if Bernoulli(}M_{G}\text{)}=0 \\ 1-x_{G} & \text{if Bernoulli(}M_{G}\text{)}=1 \end{matrix}\right.$

5. Generate environmental risk factor with misclassification: $x_{E}^{O}=\left\{\begin{matrix} x_{E} & \text{if Bernoulli(}M_{E}\text{)}=0 \\ 1-x_{E} & \text{if Bernoulli(}M_{E}\text{)}=1 \end{matrix}\right.$

6. Generate subject’s initial state: $y_{0}-1\sim\text{Bernoulli(}P\text{(}x_{\text{A}}\text{))}$, where $P(x_{A})=\alpha(x_{A}-45)\text{ }$ is the probability

that the subject developed the disease at baseline, and the value of $\alpha\text{ }$ is set to make the overall prevalence

of disease in the CLSA comprehensive cohort equals $\pi_{D}$.

**B. Generate Transition Time from Healthy to Diseased**

**Varied Parameters**

1.Weibull scale ($\lambda_{HI}$) and shape ($\rho_{HI}$) parameter for transition from Healthy to Diseased. For COPD,

$\lambda_{HI}$=70 and $\rho_{HI}$=2.5; for dementia, $\lambda_{HI}$=50 and $\rho_{HI}$=5.5; for Parkinson’s disease, $\lambda_{HI}$=125 and

$\rho_{HI}$=3.5.

2. Logarithm of hazard ratios for environmental risk factor $\beta_{E}\in(\log1,\ldots,\log3)$, genotype risk factor

$\beta_{G}\in(\log1,\ldots,\log3)$, and their interaction $\beta_{EG}\in(\log1,\ldots,\log10)$

**Fixed Parameters**

1.Weibull scale ($\lambda_{HD}$=42) and shape ($\rho_{HD}$=4) parameter for transition from Healthy to Dead

2. Loss to follow-up rate ($\lambda_{LTFU}$=0.02)

3. Length of follow-up $T_{L}=21$

**Simulation procedure**

1.Generate time to loss to follow-up $t_{LTFU}\sim\text{exponential (}\lambda_{LTFU}\text{)}$

2. Generate transition time from Healthy to Dead $t_{HD}\sim\text{Weibull}(\lambda_{HD},\rho_{HD})$ and potential transition

time from Healthy to Diseased: $t_{HI}^{P}\sim\text{Weibull} (\lambda_{HI}\exp(\beta_{E}x_{E}+\beta_{G}x_{G}+\beta_{EG}x_{E}x_{G}+F), \rho_{HI})$, where

$F\sim\text{Normal}(0,\text{ }\sigma^{2}\text{) and }\sigma=0.585$, given time is left truncated at $x_{A}$.

3. If $\min(t_{LTFU},\text{ }t_{HD},\text{ }t_{HI}^{P})=t_{HI}^{P}$, then transition time from Healthy to Diseased is observed at $t_{HI}^{P}$.

1. If $\min(t_{LTFU},\text{ }t_{HD},\text{ }t_{HI}^{P})=t_{HD}$, then transition time from Healthy to Diseased is censored at $t_{HD}$.
2. If $\min(t_{LTFU},\text{ }t_{HD},\text{ }t_{HI}^{P})=t_{LTFU}$, then transition from Healthy to Diseased is censored at $t_{LTFU}$.

**C. Generate Transition Time from Healthy to Diseased under Repeated measurements**

**Fixed Parameters**

1. $0=v_{1}<v_{2}<\text{ }\ldots<v_{8}=T_{L}$ are the 8 time points that participants are measured, i.e. subjects are measured

every 3 year for 21 years.

**Simulation procedure**

1. If $\min(t_{LTFU},\text{ }t_{HD},\text{ }t_{HI}^{P})\geq T_{L}$, then transition time is censored at $T_{L}$.
2. Else if $\min(t_{LTFU},\text{ }t_{HD},\text{ }t_{HI}^{P})=t_{HD}$, then transition time is censored at $t_{HD}$.
3. Else if $\{t_{LTFU},\text{ }t_{HD},\text{ }t_{HI}^{P}\}\in(v_{i}\text{, }v_{i+1})\text{, 1}\leq i\leq7$, then transition time is censored at $t_{HD}$.
4. Else if $v_{i}<t_{HI}^{P}\leq v_{i+1}<\min(t_{LTFU},\text{ }t_{HD})$, then transition time is observed at $v_{i+1}$.
5. Else if $\{t_{HD},\text{ }t_{HI}^{P}\}\in(v_{i}\text{, }v_{i+1})\text{ and }t_{LTFU}>v_{i+1}\text{, 1}\leq i\leq7$, then transition time is censored at $t_{HD}$.
6. Else if $\{t_{LTFU},\text{ }t_{HI}^{P}\}\in(v_{i}\text{, }v_{i+1})\text{ and }t_{HD}>v_{i+1}\text{, 1}\leq i\leq7$, then transition time is censored at $v_{i+1}$.
7. Else if $v_{i}<t_{LTFU}\leq v_{i+1}<\min(t_{HI}^{P},\text{ }t_{HD})$, then transition time is censored at $v_{i+1}$.
8. Else if $\{t_{HD},\text{ }t_{LTFU}\}\in(v_{i}\text{, }v_{i+1})\text{ and }t_{HI}^{P}>v_{i+1}\text{, 1}\leq i\leq7$, then transition time is censored at $t_{HD}$.

**C. Generate Transition Time from Healthy to Diseased under Repeated measurements**

**Fixed Parameters**

1. $0=v_{1}<v_{2}<\text{ }\ldots<v_{8}=T_{L}$ are the 8 time points that participants are measured, i.e. subjects are measured

every 3 year for 21 years.

**Simulation procedure**

1. If $\min(t_{LTFU},\text{ }t_{HD},\text{ }t_{HI}^{P})\geq T_{L}$, then transition time is censored at $T_{L}$.
2. Else if $\min(t_{LTFU},\text{ }t_{HD},\text{ }t_{HI}^{P})=t_{HD}$, then transition time is censored at $t_{HD}$.
3. Else if $\{t_{LTFU},\text{ }t_{HD},\text{ }t_{HI}^{P}\}\in(v_{i}\text{, }v_{i+1})\text{, 1}\leq i\leq7$, then transition time is censored at $t_{HD}$.
4. Else if $v_{i}<t_{HI}^{P}\leq v_{i+1}<\min(t_{LTFU},\text{ }t_{HD})$, then transition time is observed at $v_{i+1}$.
5. Else if $\{t_{HD},\text{ }t_{HI}^{P}\}\in(v_{i}\text{, }v_{i+1})\text{ and }t_{LTFU}>v_{i+1}\text{, 1}\leq i\leq7$, then transition time is censored at $t_{HD}$.
6. Else if $\{t_{LTFU},\text{ }t_{HI}^{P}\}\in(v_{i}\text{, }v_{i+1})\text{ and }t_{HD}>v_{i+1}\text{, 1}\leq i\leq7$, then transition time is censored at $v_{i+1}$.
7. Else if $v_{i}<t_{LTFU}\leq v_{i+1}<\min(t_{HI}^{P},\text{ }t_{HD})$, then transition time is censored at $v_{i+1}$.
8. Else if $\{t_{HD},\text{ }t_{LTFU}\}\in(v_{i}\text{, }v_{i+1})\text{ and }t_{HI}^{P}>v_{i+1}\text{, 1}\leq i\leq7$, then transition time is censored at $t_{HD}$.
